# Model Development for Fat Mass Assessment Using Near-Infrared Reflectance in South African Infants and Young Children Aged 3–24 Months

**DOI:** 10.3390/s21062028

**Published:** 2021-03-12

**Authors:** Alexander Miller, Jacqueline Huvanandana, Peter Jones, Heather Jeffery, Angela Carberry, Christine Slater, Alistair McEwan

**Affiliations:** 1 School of Electrical and Information Engineering, University of Sydney, Darlington, NSW 2008, Australia; j.huvanandana@gmail.com (J.H.); peter.jones@sydney.edu.au (P.J.); heather.jeffery@sydney.edu.au (H.J.); angela.carberry@sydney.edu.au (A.C.); alistair.mcewan@sydney.edu.au (A.M.); 2 Sydney School of Public Health, University of Sydney, Darlington, NSW 2006, Australia; 3 Independent Consultant, Maryport CA15, UK; slater_christine@yahoo.co.uk

**Keywords:** near-infrared reflectance (NIR), fat mass (FM), deuterium dilution (DD), dual energy X-ray absorptiometry (DXA)

## Abstract

Undernutrition in infants and young children is a major problem leading to millions of deaths every year. The objective of this study was to provide a new model for body composition assessment using near-infrared reflectance (NIR) to help correctly identify low body fat in infants and young children. Eligibility included infants and young children from 3–24 months of age. Fat mass values were collected from dual-energy x-ray absorptiometry (DXA), deuterium dilution (DD) and skin fold thickness (SFT) measurements, which were then compared to NIR predicted values. Anthropometric measures were also obtained. We developed a model using NIR to predict fat mass and validated it against a multi compartment model. One hundred and sixty-four infants and young children were included. The evaluation of the NIR model against the multi compartment reference method achieved an r value of 0.885, 0.904, and 0.818 for age groups 3–24 months (all subjects), 0–6 months, and 7–24 months, respectively. Compared with conventional methods such as SFT, body mass index and anthropometry, performance was best with NIR. NIR offers an affordable and portable way to measure fat mass in South African infants for growth monitoring in low-middle income settings.

## 1. Introduction

Inexpensive and accessible body composition measurement represents an important step in addressing global development and nutritional needs. Currently, the assessment of an infants’ nutritional status is mainly based on measures of growth centred around weight and length percentiles for a given age, with the widespread use of global standard charts from the World Health Organization (WHO) and the INTERGROWTH 21st study for birth up to 5 years of age and for pre-term newborns, respectively [1,2]. The findings of this study led to classifications of small, appropriate and large for gestational age infants, as demarcated most commonly by the 10th and 90th weight percentiles [2]. Although weight-for-age offers an understanding of infant growth to a certain extent, it is not sensitive to stunted growth and thus does not capture infants who fail to reach linear growth potential [3]. Weight-for-length can be used as an alternative proxy for growth, although accurate length measurements can be problematic, especially in infants. Another commonly used anthropometric measure is mid-upper arm circumference (MUAC), which often guides assessments of wasting [4]. Although MUAC is easily obtained, its reliability and the relationship to mortality in the infant populations are not well-characterised.

A 2010 United Nations International Children’s Emergency Fund (UNICEF)/WHO report identified undernutrition as a contributing cause in over one third of child deaths [5]. The major proportion of these deaths occur within the first year of life, particularly in the neonatal period and in low-middle income settings [6]. Malnutrition, including both under and over nutrition, has also shown impacts beyond infancy, with links to diabetes and obesity in later life [7]. Undernutrition in early life can potentially lead to brain dysfunction and reduced cognitive development [8,9]. Body fat mass (FM) has been recognized to be closely associated with undernutrition in neonates [7].

There are various approaches to determine fat mass in infants, such as dual-energy X-ray absorptiometry (DXA), air-displacement plethysmography (ADP), deuterium dilution (DD), and multi compartment models. A four-compartment (4C) model is considered the criterion method for body composition assessment [10]. For infants and young children, the 4C-model described by Butte et al. [10] includes the assessment of total body water by DD, the assessment of bone mineral content by DXA and the assessment of total body potassium (TBK) by potassium counting. DD is the reference method for measuring total body water (TBW), from which fat-free mass (FFM) is derived. FM is the difference between body weight and FFM. DXA is the reference method for assessing bone mineral content but can also be used to obtain an estimate of total body adipose tissue. Potassium counting measures the amount of naturally occurring potassium-40 in the body. This only occurs within cells and is a measure of body cell mass, which is related to muscle mass. Equipment for measuring TBK in infants and young children is not widely available, but TBK can be predicted using the equations of Fomon and Nelson [11]. The reference methods involve the use of large pieces of equipment (DXA and potassium-40 counters) or laboratory analysis of samples collected in the field, but simpler methods are required for the routine monitoring of body composition to assess changes as children grow. The multi-compartment approach involves fewer assumptions about body composition than DXA or DD predicted fat mass values, and is therefore regarded as the preferred method for the validation of new methods of assessing body composition.

Anthropometric approaches may be suitable for use in low- and middle-income settings, given their simplicity and scalability. Relevant metrics include birthweight, weight-for-length or body mass index (BMI: weight-for-length-squared) and their corresponding percentiles. Though important measures, work by Carberry et al. has demonstrated that measures of body fat percentage (BF%) may be more predictive in identifying a composite neonatal morbidity compared with birthweight percentiles [3]. Mid-upper arm circumference (MUAC) may also be used in screening for acute malnutrition, though there are limited data on its reliability and predictive value for mortality in infants under 6 months [12].

As over fifty percent of under five deaths occur in the neonatal period (first 28 days) and early infancy and the majority of these deaths are in low- and middle-income countries [6], there is a clear need for a simple, low-cost and portable method for the rapid assessment of body composition. Near-infrared (NIR) reflectance has previously been explored and used as an input to body composition assessment, with several studies evaluating the agreement between commercially-available devices operating on these principles with other reference methods [12,13] and the development of models surrounding body composition [14]. NIR has been shown to have the potential to offer the low cost, portable method that is needed.

The near-infrared (NIR) region of the electromagnetic spectrum spans from approximately 600–1300 nm, where the absorption properties of tissue vary according to its constituents. These include water, fat and collagen. Early studies of NIR spectrometry by Conway et al. [15] identified absorption peaks in the spectral profile measured from the triceps site of adult subjects, corresponding to the pure fat absorption band at 930 nm and pure water absorption band at 970 nm [15]. A more recent study published in 2013 by Jacques is also in agreement with these absorption bands [16]. However, a 2015 study by Wilson et al. has determined the absorption peaks to be higher in the 1100–1900 range [17]. While it is quite possible that this spectrum range could provide superior results, it would be more impractical for an affordable field device. Light emitting diodes (LEDs) begin to at least double in price when reaching 1050 nm. It is expected for the device to have at least four LEDs. In the 850–1050 nm range, most LEDs cost between USD 1–10, while from 1100–1800 nm prices can range from USD 20 to USD 130. This could take the device from being less than USD 50 to being over USD 100, which starts to become too steep of a price for developing countries.

Building on this work, the primary aim of this paper was to develop a model using features extracted from NIR interactance for the estimation of infant/young child fat mass. The second aim was to compare the performance of this model against other accessible methods of body composition assessment for low- and middle-income countries, using the multi compartment model as the evaluation method. The comparative measures for adiposity and/or nutritional status include mid-upper arm circumference, weight-for-length percentiles, weight-for-age percentiles, length-for-age percentiles, body mass index (BMI), and sum of skinfolds.

## 2. Materials and Methods

### 2.1. Study Population

Infants and young children from birth (within first 48 h) up to 24 months of age were recruited from maternity and follow-up clinics at the Developmental Pathways for Health Research Unit (DPHRU) at the Chris Hani Baragwanath Hospital, Soweto, South Africa. The hospital serves an urban poor area with a predominantly African black population. Recruitment took place over a period of 9 months (April to December 2016). Inclusion criteria included well infants born at term (37–41 weeks) with an absence of major congenital anomalies, morbidity and chronic health problems. Other inclusion criteria included ≤24 months of age at measurement, mother’s age > 18 years, singleton pregnancy, living within the study area as well as English literacy and comprehension. Infants that had undergone any DXA scans in the prior 12 months were excluded from enrolment. Informed parental written consent was obtained and participation was voluntary.

### 2.2. Ethics

This study was approved by the Ethics Committees at the University of Sydney (USyd) and the University of Witwatersrand (Wits) (USyd HREC number: Project No.: 2015/595; Wits HREC number: M150774).

The study has also been registered on the Australia and New Zealand Clinical Trials Registry (ANZCTR) number: ACTRN12615001318572.

### 2.3. Data Collection

The data in this study were collected as part of a population-based cross-sectional validation study. Data collected included anthropometric measures such as weight, length, head and mid-upper arm circumferences, and skinfold thickness. Weight was measured using electronic baby scales (Seca 376, Hamburg, Germany). Length was measured using an infantometer (Harpenden, Holtain Model 702), which had a fixed headboard and moveable footboard following a two-trained person technique. Head, abdomen, thigh and mid-upper arm circumferences were measured using a metal tape measure. Skinfold measurements were conducted using handheld callipers at four anatomical sites: triceps, subscapular, mid-thigh and flank. Other metadata such as sex and ethnicity were also included.

Total body fat percent (BF%) was measured using DXA (Hologic Discovery DXA S/N 86254 APEX software version 4.0.2 Hologic Inc., Waltham, MA, USA) with relevant paediatric software installed. The DXA measurement involved the infant lying flat on a scanner bed to measure bone mineral content (BMC) and BF%. Bone mineral content information was collected to serve as an input for the final compartment model [10,11].

**Figure 1:**
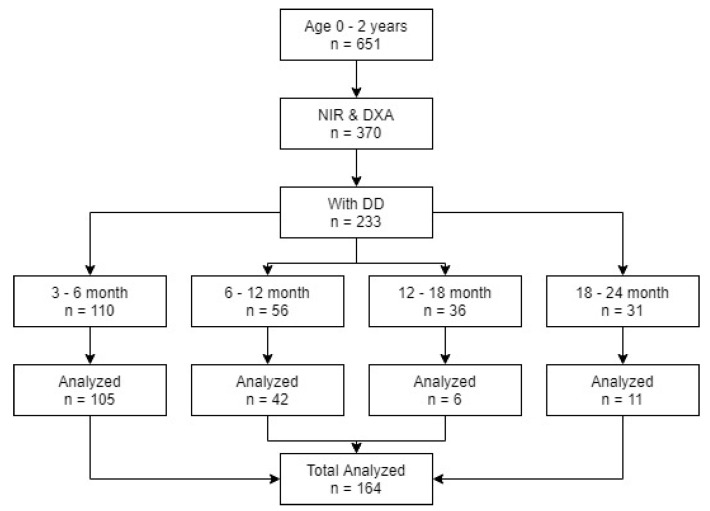
Recruit flow chart.

Body composition was also assessed by deuterium dilution. This involved the infant receiving a carefully weighed dose (approximately 1 g) of deuterium oxide (99.8 atom % D, Sercon Ltd., Crewe, UK) using an oral dosing syringe, which was weighed on an analytical balance accurate to 0.0001 g. The weight of D_2_O consumed by the infant was determined based on the difference in syringe weight and contents before and after dose administration. A basal saliva sample was collected before this dose was given and then two further saliva samples were collected after the dose had equilibrated with the infants’ body water, with the final sample at 3 h after the dose was administered. The enrichment of deuterium in saliva samples was analysed using a Fourier transform infrared spectrometer (Agilent 4500 Series FTIR, Santa Clara, CA, USA) [18]. TBW (kg) was calculated from the dilution space and was used in the final multi-component model. FFM (kg) was estimated from TBW using age and sex specific hydration factors. FM (kg) is the difference between body weight and FFM [18]. FM was expressed as a percentage of the body weight (BF%). Deuterium dilution data were rejected if the apparent deuterium content of the basal sample was high (possibly due to spectral interference due to the presence of an interfering compound in the saliva), if the deuterium enrichment was lower than expected, resulting in very low (non-physiological) values of BF% (<10%), probably due to loss of deuterium in dribbled saliva. Partway through the study a change in personnel performing the deuterium analysis resulted in a loss of data, which can be seen in the drop of subjects in Figure 1. Mothers of infants were exclusively breastfeeding during the study, meaning that giving any other fluids by mouth was not accepted. As a result of this, all infants from 0–3 months did not have body composition assessed by deuterium dilution.

**Figure 2:**
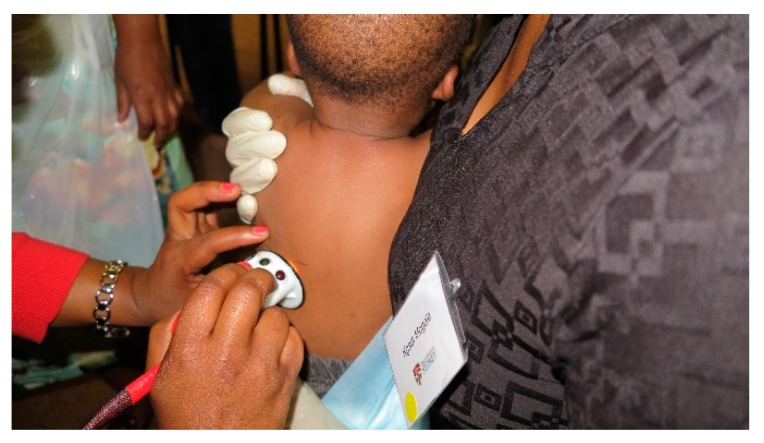
NIR measurement conducted on the subscapular site using the Ocean Optics QE Pro NIR Device.

Prior to NIR measurements, reference scans against ambient light and a dark material were obtained. The spectrometer (QEPro-FL, Ocean Optics, Orlando, FL, USA) collected a range of wavelengths from 350 to 1100 nm. The reflection profiles were determined by customised software (QEProInterface v3.1) which was developed in-house using LabVIEW (National Instruments, v2014), adjusting the measured intensity for the dark and light calibration scans. The NIR measurements were conducted through the skin’s surface at the same four anatomical locations where skinfold thickness measurements were obtained, triceps, subscapular, mid-thigh and flank, for approximately 30 s performed by a researcher (Figure 2).

Research assistants were rigorously trained for accuracy and reproducibility of anthropometry measurements by experienced anthropometrists, who had participated in the WHO Multicentre Growth Reference Study [1]. Two standardisation exercises were conducted during the data collection period. In addition, specifically for this project, a four station SCORPIO method of skills training [19] was conducted, which included stations addressing: how to set up the NIR device for daily testing and how to conduct the Quality Control (QC); correct NIR probe positioning and skinfold measurements; accurate anthropometric measurements and data entry; how to check that NIR data are reading correctly, and troubleshooting. All anthropometric and NIR measurements were conducted in duplicate by two trained researchers. Any pair of measurements falling outside the maximum allowed differences were repeated by both researchers. If this second pair of measurement values again exceeded the limits for the measurement, the researchers repeated the measurement for a third and final time.

### 2.4. Model Development

Pre-processing, model development and statistical analysis were completed using Python (Python Software Foundation, version 3.6, https://www.python.org/, accessed on 1 August 2017).

Feature selection and parameter fitting were undertaken on a randomised division (three fifths) of the dataset and subsequently evaluated on the remaining two fifths. The division was stratified based on age and sex, that is, similar distributions of ages and sex across the training and testing subsets. The model development process involved two stages: first, the feature selection, and the second, model fitting on a subset (training and validation set) of the available data.

The wavelength range of interest spanned 850–1100 nm, with potential features being sampled from 10 nm intervals and expressing the ratio between them as a percentage. This resulted in a set of 300 features for each measurement site (subscapular, flank, triceps and thigh). Using this feature set, we then selected up to two features along with weight/length ratio. Using a leave-one-out cross-validation, the mean estimation error was determined for each feature. This process involves fitting all except one sample from the training set to the target variable and evaluating the excluded sample, with a repeat of this process for every possible sample. By taking the features with the smallest mean estimation error, model fitting was subsequently undertaken.

The NIR model was developed using the multi compartment model as the reference. The model was subsequently evaluated by its agreement and correlation with fat mass measured by the same multi compartment model. The multi compartment model was based on that adopted by Fomon et al. [11] and Butte et al. [10] and can be seen in Equation (1) below.

(1) $$\mathrm{FFM}=\frac{1009.4108*\mathrm{TBW}+1000*\mathrm{BMC}}{994-2.87851{*\mathrm{TBK}}_{\mathrm{FFM}}}$$

where total body water (TBW), bone mineral content (BMC), and fat-free mass (FFM) are in Kg. The total potassium (${\mathrm{TBK}}_{\mathrm{FFM}}$) is in mEQ/kg FFM, where mEQ is the nitrogen to potassium ratio, and estimated for male and female infants using Equations (2) and (3), respectively, where mo is age in months. As a potassium counter was not available, these were based on polynomial curve fits to the table for TBK (mEQ/kg) from Fomon et al. [11].

(2) $$\mathrm{TBK}=0.0009{\mathrm{mo}}^{3}-0.0505{\mathrm{mo}}^{2}+1.1047\mathrm{mo}+49.108$$

(3) $$\mathrm{TBK}=0.0011{\mathrm{mo}}^{3}-0.0629{\mathrm{mo}}^{2}+1.2957\mathrm{mo}+49.113$$

## 3. Results

Of the 651 eligible term infants (37–42 weeks gestational age) aged from birth (48 h) up to 24 months, 416 (63.9%) had available DXA measurements. Of these, 370 infants (88.9%) had complete NIR and anthropometric measurements. Of these, 164 infants (25.66%) aged from 3 to 24 months had acceptable deuterium dilution measurements and were included in the model development (Figure 1). As the multi compartment model used for reference and evaluation uses both DD and DXA predicted values, our total population was 164.

The characteristics of the study population are summarised in Table 1. Weight, length, fat mass, and bone mineral density all increase as a newborn ages and this is to be expected. Because of both length and weight increasing, there is not much difference in skinfold measurements. It is normal for there to be little fluctuation in a small age range. A one-way ANOVA with alpha 0.05 was conducted to determine if there were statistically significant differences between age groups for mid-upper arm circumference and subscapular, flank, thigh, and triceps skinfolds. For the subscapular skinfold, there were no statistically significant differences between group means as determined by one-way ANOVA (F(2, 325) = 1.519, *p* = 0.22). Flank, thigh, and triceps skinfolds all also had no statistically significant differences between group means with the following one-way ANOVA results, respectively: (F(2, 325) = 0.6710, *p* = 0.51), (F(2, 325) = 0.5431, *p* = 0.58), and (F(2, 325) = 0.053, *p* = 0.95). Mid-upper arm circumference, however, did report a statistically significant difference between groups as determined by one-way ANOVA (F(2, 325) = 9.330, *p* = 0.0001). A Tukey–Kramer post-hoc test was then performed for the MUAC dataset. The q statistic between the 3–24 month and 7–24 month groups was 4.19, which is greater than the critical value of 3.335, so the null hypothesis is rejected and there is a statistically significant difference between the two. The q statistic between the 7–24 month and 3–6 month groups was 6.11, which is also greater than the critical value and once again the null hypothesis is rejected and there is a statistically significant difference. The q statistic between the 3–24 month and 3–6 month groups was 2.86, which is less than the critical value, meaning the null hypothesis is not rejected and there is no statistically significant difference between the groups.

The acceptability of deuterium dilution measurements was determined using multiple quality control checks, including the reproducibility of deuterium analysis, the equivalence of the two post dose saliva samples, and whether the calculated TBW was within a normal range (45–70% body weight). Data from participants with TBW outside the normal range were rejected. High TBW caused by lower than expected deuterium enrichment results in very low (non-physiological) values of BF%, probably due to a loss of deuterium during or just after dosing due to dribbling. This can be seen in Figure 1, where the number of children who received D20 and the number of children used in the model are different.

Table 2 summarises the wavelengths selected from the recursive feature selection process for the model using length. Linear coefficients are based on the regression model fitted to the training set. Equation (4) shows the model equation with reflection ratios and weight-for-length (g/cm). Note that reflection values are expressed as a percentage of reflected light at the given wavelength, where R_1–4_ refer to different wavelengths. Length was removed for some of the results.

(4) $$\mathrm{FM}=\beta_{1}\frac{R_{1}}{R_{2}}+\beta_{2}\frac{R_{3}}{R_{4}}+\beta_{3}\frac{W}{L}+\;\varepsilon$$

**Figure 3:**
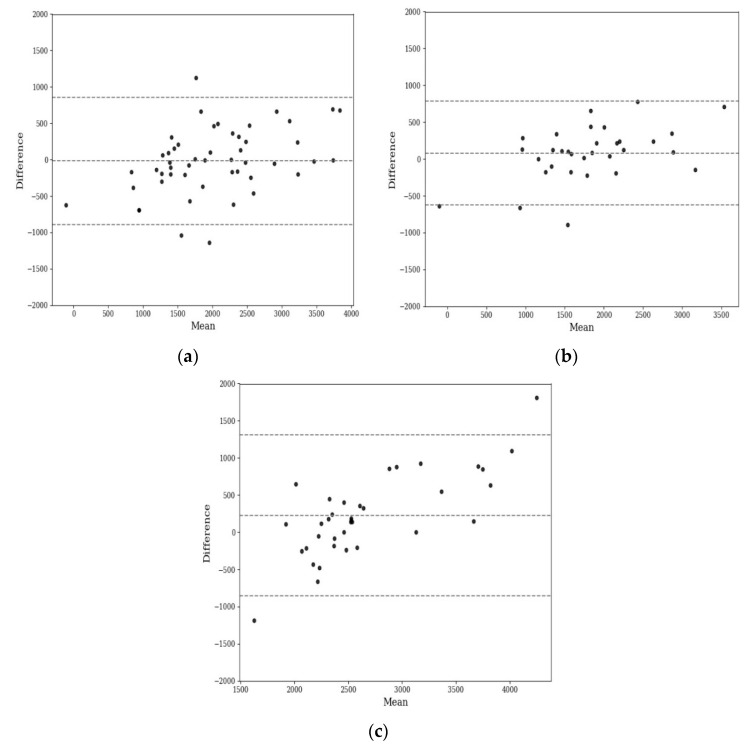
Bland–Altman plot for fat mass agreement between NIR (D2O as reference method) and the 4-compartment model for (**a**) all ages N = 164, (**b**) 0–6 months N = 105, and (**c**) 7–24 months N = 59.

Model evaluation and comparison with other anthropometric methods was undertaken on the remaining 40% (testing set) of the data. Correlation and Bland–Altman agreement for the models are summarised in Table 3, with Bland–Altman plots for the model displayed in Figure 3. The linear correlation observed in the Bland–Altman plots is likely due to a combined effect of bias (as the mean difference is above zero) and the difference in standard deviation between methods [20].

The agreements of NIR model measurements with fat mass evaluated by the multi-compartment model were −78.9 (−954.3, 796.6), 7.6 (−701.3, 716.6), and 174.5 (−914.7, 1263.6) for 3–24 months, 0–6 months, and 7–24 months, respectively, the first number being the average predicted fat mass for all subjects in the test set. The numbers in parentheses are the agreement interval, within which 95% of the differences to the evaluation method fall. With length not included, the agreements were −105.8 (−992.2, 780.7), 42.0 (−765.5, 849.5), and 194.6 (−846.9, 1263.1) for 3–24 months, 0–6 months, and 7–24 months, respectively.

## 4. Discussion

### 4.1. Estimation of Fat Mass and Comparison with Other Models

This study focused on the comparison of NIR interactance-based models for the estimation of body fat in infants and young children aged 3–24 months against other accessible methods for low-middle income settings. These models were evaluated based on Bland–Altman agreement with DXA-derived and DD-derived FM, the variance explained by each of the models measured by R and the distribution of standard error across 100 repetitions of cross-validation.

Across all age groups (3–24 months, 0–6 months, and 7–24 months), NIR displayed narrower 95% limits of agreement from Bland–Altman analysis than all other methods except deuterium dilution. Even with length removed, NIR was near the top for highest correlation, while also having the lowest limits of agreement. Deuterium dilution showed a very strong correlation, which may be due to it having a large influence in the multi compartment model. TBW represents almost 60% of total body weight in infants. DXA, despite being regarded as a reference method for body composition assessment, had fairly poor correlation, being below SFT for all age groups. The cost, portability, and requirement of a highly trained professional for DD and DXA hinder their use in low- and middle-income settings as well.

The WHO percentiles, based on weight and length, had very large limits of agreement and poor correlation. These percentiles do not predict fat mass, but are used to give an overall assessment of nutritional status. While MUAC had higher correlation, it still did not show great results for the predictability of fat mass.

BMI performed fairly well in the 3–6 month age group but very poorly in older ages. Like the percentiles for length-for-age and weight-for-length, length requires an appropriate length board with a moving foot piece and trained staff for accurate measurement. Weight was shown to not be a strong indicator of fat mass.

The sum of skinfold thickness often displayed the next highest agreement and correlation with 4C for fat mass predictions. The Slaughter equation (Equations (5) and (6) for male and female infants, respectively) was used for the sum of skinfold thickness [21].

(5) $${\mathrm{BF}\%}_{M}=1.21\;\times \;\sum \mathrm{SFT}-0.008\;\times \;\sum {\mathrm{SFT}}^{2}-1.7$$

(6) $${\mathrm{BF}\%}_{F}=1.33\;\times \;\sum \mathrm{SFT}-0.013\;\times \;\sum {\mathrm{SFT}}^{2}-2.5$$

where ∑SFT is based on the sum of subscapular and triceps skinfold thicknesses in mm. The BF% was multiplied by total weight to obtain fat mass. However, it is also necessary to consider operator training requirements when comparing the methods. SFT measurements generally require a high degree of training and are conducted in duplicate with defined tolerance to ensure agreement. There is also a tendency for skinfold thickness measurements to overestimate fat in lean infants and underestimate it in those with more fat [22]. SFT measurements, as with the other anthropometric measurements, were performed by highly trained staff.

The NIR model with length included performed better in almost all age groups. Even though the length can be difficult to measure in young infants, having the additional information helps the model train for more accurate results. If an accurate length board measurement is not readily accessible [23], it is possible to use the model without length and not see a drop greater than 0.1 in correlation.

### 4.2. Wavelength Selection

The wavelength range for feature selection spanned 850–1100 nm. Recursive feature elimination identified all wavelengths in the range that were relevant for the fat mass estimation model. This range encompassed peaks of water (930 nm) and fat (970 nm) reported in the literature [15]. In a study by Conway, J.M. et al., wavelength ratios were used in order to remove and normalise the baseline offset [15]. This idea for ratios was used in developing our model.

### 4.3. Strengths and Limitations

One of the strengths of this study is the large population-based sample across a wide range of age groups (3 months–2 years). We also used the multi compartment model—one of the gold standard methods for body composition—as the reference and evaluation method. However, in the most commonly used 4-Compartment models, body volume measurements are required, but these are not possible in infants between 6 months and 2 years so a mass-based multi compartment model was adopted. As a potassium counter was not available, assumptions for potassium had to be made. This could lead to some limitations of its use. As deuterium dilution was only performed on infants older than 3 months, we have no results for 0–3 months, which is a significant limitation.

The model development for the estimation of body fat in infants aged 3–24 months was limited by the non-normal distributions of the infant data collected, as well as the relatively low representation of “low fat” infants, with Z-score plots on WHO percentile charts indicating skewness towards the higher end across skinfolds.

The model was compared with DD and DXA, which are considered two criterion methods for measuring body composition.

One of the limitations is the lack of reference data for body composition in infants and young children. Most published data come from high-income countries and were collected when breastfeeding was not very common [24]. This affected our ability to determine if a child was on the higher or lower end for body fat percentage for their age.

Anthropometric indicators are not strong indicators of body composition on their own accord, which is why there is a crucial need for a simple to use device to estimate body composition. However, obtaining a body composition result is only one issue in preventing/intervening in the malnutrition of infants and young children. Once a body composition value is obtained, the interpretation of the results for clinical or public health actions is still limited as there are no body composition reference data across populations, although Butte et al. published longitudinal data for 0–2 year old children in the United States of America [10]. This limitation is especially evident in our study, conducted in Soweto. Soweto is a low-income area with a predominately black population. There is very little information about the body composition of this population as most studies are conducted in middle- to high-income countries with predominantly white populations.

## 5. Conclusions

This study developed and evaluated models using near-infrared interactance features in comparison to other portable and accessible methods for infant body composition assessment in low-middle income settings. The NIR models offered a more robust means of fat mass estimation in infants aged 3–24 months, showing a higher correlation and narrower limits of agreement than other low cost methods. NIR is a solution to a chronic need by providing an affordable and portable device to measure fat mass in infants and young children. There is a large amount of future work that can be accomplished from this study. For instance, it is worth looking into seeing if any of the measurements in Table 1 have a large effect on fat mass in infants aged 3–24 months. The different methods used to predict fat mass can also be compared to discover the strengths and weaknesses of various models. Further steps that need to be taken are conducting a study where air displacement plethysmography equipment is available to obtain results on infants aged 0–3 months. A study also needs to be conducted where the NIR device is tested in a low-fat population.

## Figures and Tables

**Table 1:** Of enrolled infants aged 3–24 months, 416 (63.9%) had available DXA measurements. Of these, 370 (88.9%) had complete NIR and anthropometric measurements. Of these, 164 (25.66%) had acceptable deuterium dilution measurements.

Variable	3–24 Months	3–6 Months	7–24 Months
n	164	105	59
Age (months)	7.1 ± 5.7	3.6 ± 1.5	13.3 ± 5.2
Male (%)	48.17	50.48	44.07
Weight (g)	7561 ± 2015	6437 ± 1231	9562 ± 1530
Length (cm)	65.8 ± 8.6	60.8 ± 4.3	74.8 ± 6.7
Fat mass (g) (DD)	2073 ± 878	1738 ± 677	2668 ± 880
Adipose Tissue Mass (g) (DXA)	2146 ± 861	1935 ± 797	2523 ± 843
Fat mass (g) (4C)	2117 ± 881	1783 ± 699	2712 ± 858
Total Body Water (kg) (TBW)	4.4 ± 1.1	3.8 ± 0.6	5.4 ± 0.9
Bone Mineral Density	0.15 ± 0.05	0.12 ± 0.03	0.20 ± 0.04
Mid-upper arm circumference (cm)	14.2 ± 2.3	13.6 ± 2.0	15.2 ± 2.4
Subscapular skinfold (cm)	9.3 ± 2.1	9.5 ± 1.9	8.9 ± 2.3
Flank skinfold (cm)	11.0 ± 2.9	11.2 ± 2.8	10.7 ± 3.0
Thigh skinfold (cm)	19.9 ± 3.8	20.1 ± 3.6	19.5 ± 4.0
Triceps skinfold (cm)	9.6 ± 1.8	9.5 ± 1.7	9.6 ± 2.0

Footnote: Values are Mean and ± Standard Deviation.

**Table 2:** Model wavelengths and coefficients. Scan locations are shown with age groups and wavelengths are in nm as shown in parentheses.

Coefficient	Estimate		
	3–24 Months (Subscapular)	3–6 Months (Subscapular)	7–24 Months (Flank)
$\beta_{1}$ Wavelength ratio	−300.58 (930/970)	−270.03 (930/970)	−52.80 (910/1090)
$\beta_{2}$ Wavelength ratio	742.77 (950/960)	775.13 (950/960)	208.03 (990/1000)
$\beta_{3}$ Anthropometric ratio	36.53 (W/L)	34.69 (W/L)	28.74 (W/L)
ε	−46,818.48	−53,255.32	−16,176.06

**Table 3:** Fat mass prediction for each method analysed: Overview of correlation and Bland–Altman agreement. Methods for comparison include body mass index (BMI), sum of skinfold thicknesses (SFT), weight-for-length percentile (W/L), length-for-age percentile (L/A), weight-for-age percentile (W/A), mid upper arm circumference (MUAC), Deuterium Dilution (DD), and dual-energy X-ray absorptiometry (DXA). The training set fit, including correlation (R) and Bland–Altman agreement, is shown. Independent Testing set R and agreement are also included. Bland–Altman agreement is displayed as mean difference (95% confidence intervals). Training was performed with the 4-Compartment model as the target and was subsequently evaluated with the 4-Compartment model. The table is arranged from best to worst test R for each age group.

	Target	Model	Train R	Train Bland–Altman	Test R	Test Bland–Altman
Fat Mass (grams)	3–24 Months N = 164	DD	1.000	61.6 (1.1, 122.0)	0.913	5.5 (−752.3, 763.4)
		NIR (No L)	0.798	0.0 (−1002.6, 1002.6)	0.895	−105.8 (−992.2, 780.7)
		NIR	0.830	0.0 (−927.5, 927.5)	0.885	−78.9 (−954.3, 796.6)
		SFT	0.810	782.6 (−264.2, 1829.4)	0.824	670.9 (−592.7, 1934.5)
		MUAC	0.531	0.0 (−1408.8, 1408.8)	0.745	−106.7 (−1649.7, 1436.3)
		DXA	0.747	−42.9 (−1260.7, 1174.8)	0.657	2.3 (−1424.7, 1429.4)
		W/A	0.609	0.0 (−1318.3, 1318.3)	0.589	−12.2 (−1529.6, 1505.3)
		BMI	0.429	0.0 (−1501.8, 1501.8)	0.545	−90.1 (−1669.0, 1488.7)
		W/L	0.291	0.0 (−1590.8, 1590.8)	0.303	−139.6 (−1903.3, 1624.1)
		L/A	0.209	0.0 (−1625.9, 1625.9)	0.125	−118.5 (−1956.4, 1719.3)
	3–6 months N = 105	NIR	0.792	0.0 (−760.7, 760.7)	0.904	7.6 (−701.3, 716.6)
		NIR (No L)	0.800	0.0 (−747.3, 747.3)	0.877	42.0 (−765.5, 849.5)
		DD	0.999	72.9 (24.5, 121.2)	0.813	−19.7 (−961.2, 921.9)
		SFT	0.790	599.0 (−225.1, 1423.0)	0.794	604.4 (−550.2, 1759.0)
		BMI	0.542	0.0 (−1047.8, 1047.8)	0.790	25.1 (−1040.5, 1090.6)
		DXA	0.721	−109.8 (−1087.8, 868.2)	0.768	−243.5 (−1472.8, 985.8)
		W/A	0.695	0.0 (−895.7, 895.7)	0.663	53.4 (−1159.1, 1265.8)
		MUAC	0.463	0.0 (−1104.7, 1104.7)	0.629	117.2 (−1276.2, 1510.7)
		W/L	0.328	0.0 (−1177.6, 1177.6)	0.449	60.7 (−1419.1, 1540.5)
		L/A	0.255	0.0 (−1205.4, 1205.4)	0.192	146.2 (−1433.9, 1726.3)
	7–24 months N = 59	DD	1.000	51.4 (−17.7, 120.5)	0.999	39.5 (−27.7, 106.7)
		NIR	0.625	0.0 (−1278.0, 1278.0)	0.818	174.5 (−914.7, 1263.6)
		NIR (No L)	0.587	0.0 (−1326.0, 1326.0)	0.793	194.6 (−846.9, 1236.1)
		SFT	0.603	877.9 (−428.8, 2184.5)	0.763	1097.3 (−55.0, 2249.5)
		DXA	0.542	23.9 (−1825.3, 1873.1)	0.702	291.2 (−915.2, 1497.6)
		MUAC	0.473	0.0 (−1442.9, 1442.9)	0.649	288.0 (−1238.9, 1814.9)
		W/A	0.639	0.0 (−1259.0, 1259.0)	0.582	181.7 (−1194.1, 1557.6)
		W/L	0.526	0.0 (−1393.0, 1393.0)	0.428	75.9 (−1450.7, 1602.4)
		BMI	0.512	0.0 (−1406.9, 1406.9)	0.421	169.4 (−1368.2, 1707.0)
		L/A	0.062	0.0 (−1634.4, 1634.4)	0.009	217.5 (−1473.5, 1908.5)

## Data Availability

The data presented in this study are available on request from the corresponding author. The data are not publicly available due to privacy and ethical concerns.
